# Ways of thinking in STEM-based problem solving

**DOI:** 10.1007/s11858-023-01474-7

**Published:** 2023-03-03

**Authors:** Lyn D. English

**Affiliations:** Queensland University of Technology, Brisbane, Australia

**Keywords:** Problem solving, STEM, Mathematical modelling, Critical thinking, Systems thinking, Design-based thinking, Adaptive and innovative thinking

## Abstract

This article proposes an interconnected framework, *Ways of thinking in STEM-based Problem Solving*, which addresses cognitive processes that facilitate learning, problem solving, and interdisciplinary concept development. The framework comprises critical thinking, incorporating critical mathematical modelling and philosophical inquiry, systems thinking, and design-based thinking, which collectively contribute to adaptive and innovative thinking. It is argued that the pinnacle of this framework is learning innovation, involving the generation of powerful disciplinary knowledge and thinking processes that can be applied to subsequent problem challenges. Consideration is first given to STEM-based problem solving with a focus on mathematics. Mathematical and STEM-based problems are viewed here as goal-directed, multifaceted experiences that (1) demand core, facilitative ways of thinking, (2) require the development of productive and adaptive ways to navigate complexity, (3) enable multiple approaches and practices, (4) recruit interdisciplinary solution processes, and (5) facilitate the growth of learning innovation. The nature, role, and contributions of each way of thinking in STEM-based problem solving and learning are then explored, with their interactions highlighted. Examples from classroom-based research are presented, together with teaching implications.

## Introduction

With the prevailing emphasis on integrated STEM education, the power of mathematical problem solving has been downplayed. Over two decades we have witnessed a decline in research on mathematical problem solving and thinking, with more questions than answers emerging (English & Gainsburg, 2016; Lester & Cai, 2016). This is of major concern, especially since work and non-work life increasingly call for resources beyond “textbook” problem solving (Chin et al., 2019; Krause et al., 2021). Such changing demands could not have been more starkly exposed than in the recent COVID 19 crisis, where mathematics played a crucial role in public and personal discourse, in describing and modelling current and potential scenarios, and in explaining and justifying societal regulations and restrictions. As Krause et al. (2021) highlighted, “No mathematical task we can create could be a richer application of mathematics than this real situation” (p. 88).

Modelling and statistical analyses that led the search for strategies to “flatten the curve” with minimal social or economic detriment were prominent in the media (Rhodes & Lancaster, 2020; Rhodes et al., 2020). As nations strive to rebuild their economies including grappling with crippling energy costs, advanced modelling again plays a key role (Aviso et al., 2022; Oxford Economics, 2022; Teng et al., 2022). Unfortunately, the gap between the mathematical modelling applied during the pandemic and how the public interpreted the models has been “palpable and evident” (Aguilar & Castaneda, 2021). Sadly, as Di Martino (Krause et al., 2021) pointed out regarding his nation:

*People’s reactions in this pandemic underlined the spread of a widely negative attitude toward mathematics among the adult population in Italy. We have witnessed the proliferation of strange, unscientific, and dangerous theories but also the risk of refusing to approach facts that involve math and the resulting dependence to fully rely on others when mathematics is used to justify decisions. Also, on this occasion many people showed their own fear of math and their rejection of mathematical arguments as relevant factors in justification (p. 93).*

This heightened visibility of mathematics in society coupled with a general lack of mathematical literacy sparked major questions about the repercussions for education and research (Bakker & Wagner, 2020; Kollosche & Meyerhöfer, 2021). One such repercussion is the need to reconsider perspectives on STEM-based problem solving and how different ways of thinking can enhance or hinder solutions. With reference to engineering education, Dalal et al. (2021) indicated how numerous calls for a focus on ways of thinking have largely been taken for granted or at least treated at a superficial level. As these authors pointed out, while perceived as a theoretical concept, ways of thinking “can and should be used in practice as a structure for solution-oriented outlooks and innovation” (p. 109).

**Fig. 1:**
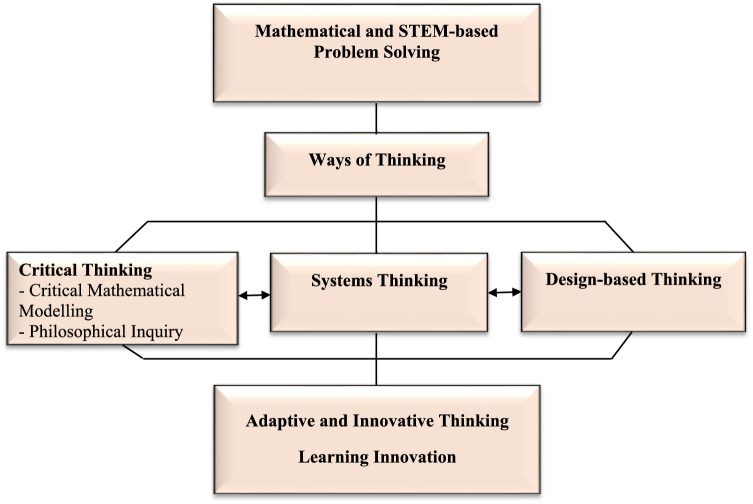
Ways of thinking in STEM-based problem solving

Given the above points, I propose an interconnected framework, *Ways of thinking in STEM-based Problem Solving* (Fig. 1), which addresses cognitive processes that facilitate learning, problem solving, decision-making, and interdisciplinary concept development (cf. Slavit et al., 2021). The framework comprises critical thinking (including critical mathematical modelling and philosophical inquiry), systems thinking, and design-based thinking. Collectively, these thinking skills contribute to adaptive and innovative thinking (McKenna, 2014) and ultimately lead to the development of learning innovation (Sect. 3.4; English, 2018).

These thinking skills have been chosen because of their potential to enhance STEM-based problem solving and interdisciplinary concept development (English et al., 2020; Park et al., 2018; Slavit et al., 2021). Highlighting these ways of thinking, however, is not denying the importance of other thinking skills such as creativity, which is incorporated within the adaptive and innovative thinking component of the proposed framework (Fig. 1), and is considered multidimensional in nature (OECD, 2022). Other key skills such as communication and collaboration (Stehle & Peters-Burton, 2019) are acknowledged but not explored here.

In line with Dalal et al. (2021), I consider the proposed ways of thinking as providing an organisational structure both individually and interactively when enacted in practice—notwithstanding the contextual and instructional influences that can have an impact here (Slavit et al., 2022). Prior to exploring these ideas, I consider STEM-based problem solving with a focus on mathematics.

## STEM-based problem solving: a focus on mathematics

Within our STEM-intensive society, we face significant challenges in promoting STEM education from the earliest grades while also maintaining the integrity of the individual disciplines (Tytler, 2016). With the increasing need for STEM skills across multiple workforce domains, contrasted with difficulties in STEM implementation in many schools (e.g., Dong et al., 2020), the urgency to advance STEM education has never been greater. With the massive disruption caused by COVID-19, coupled with problematic international relations, our school students’ futures have become even more uncertain—we cannot ignore the rapid changes that will continue to impact their lives. Unlike business and industry, where disruption creates a “force-to-innovate” approach (Crittenden, 2017, p. 14), much of school education seems oblivious to preparing students for these disruptive forces or at least are restricted in doing so by set curricula.

Preparing our students for an increasingly uncertain and complex future requires rethinking the nature of their learning experiences, in particular, the need for more relevant and innovative problems that are challenging but manageable, and importantly, facilitate adaptive learning and problem solving (McKenna, 2014). A failure to provide such opportunities may have detrimental effects on young students’ learning and their future achievements (Engel et al., 2016). Despite mathematics being cited as the core of STEM education and foundational to the other disciplines (e.g., Larson, 2017; Roberts et al., 2022; Shaughnessy, 2013), it is frequently ignored in integrated STEM activities (English, 2016; Maass et al., 2019; Mayes, 2019; Shaughnessy, 2013). For example, quantitative reasoning, which is critical to integrated STEM problem solving, is frequently “misrepresented, underdeveloped, and ignored in STEM classrooms” (Mayes, 2019, p. 113). Likewise, Tytler (2016) warned that there needs to be an explicit focus on the mathematical concepts and thinking processes that arise in STEM activities. Without this focus, STEM programs run the risk of reducing the valuable contributions of mathematical thinking. If children fail to see meaningful links between their learning in mathematics and the other STEM domains, they can lose interest not only in mathematics but also in the other disciplines (Kelley & Knowles, 2016).

Traditional notions of mathematical problem solving (e.g., Charles, 1985) are now quite inadequate when applied to our current world. At a time of increasing creative disruption, it is essential for mathematical problem solvers to be adaptive in dealing with unforeseen local, national, and international problems. Increasingly, STEM-based problems in the real world encompass more than just disciplinary content and practices. While not denying the essential nature of these components, issues pertaining to cultural, social, political, and ethical dimensions (Kollosche & Meyerhöfer, 2021; Pheasants, 2020) can also impact the solution process, necessitating the application of appropriate thinking skills. As Pheasants stressed, “If STEM education is to prepare students to grapple with complex problems in the real world, then more attention ought to be given to approaches that are inclusive of the non-STEM dimensions that exist in those problems.”

In light of the above arguments, I view mathematical and STEM-based problems as goal-directed experiences that (1) demand STEM-relevant ways of thinking, (2) require the development of productive and adaptive ways to navigate complexity, (3) enable multiple approaches and practices (Roberts et al., 2022), (4) recruit interdisciplinary solution processes, and (5) facilitate growth of learning innovation for all students regardless of their background (English, 2018). In contrast to traditional expectations, such problems need to embody affordances that facilitate learning innovation, where all students can move beyond their existing competence in standard problem solving and be challenged to generate new knowledge in solving unanticipated problems. Even students who achieve average results on standardised tests display conceptual understanding and advanced mathematical thinking not normally seen in the classroom—especially when current common practices emphasise number skills at the expense of problem solving and reasoning with numbers (Kazemi, 2020).

Limited attention, however, has been paid to how problem experiences can be developed that press beyond basic content knowledge (Anderson, 2014; Li, Schoenfeld et al., 2019), encompass the STEM disciplines, and develop important ways of thinking. In their recent article, Slavit et al. (2021) argued that STEM education should be “grounded in our knowledge of how students think in STEM-focused learning environments” (p. 1), and that fostering twenty-first-century skills is essential. Yet, as these authors highlighted, there is not much research on STEM ways of thinking, with even fewer theoretical perspectives and frameworks on which to draw. In the next section, I consider these ways of thinking, defined in Table 1, and provide examples of their applications to STEM-based problem solving.

**Table 1:** Ways of thinking in STEM-based problem solving

Critical thinking	Critical thinkers effectively evaluate and judge problem situations including statements, claims, and propositions; they analyse and reflect on solution approaches and conclusions drawn
Critical mathematical modelling	Critical mathematical modelling involves developing conceptual innovations in response to real-world needs; effective modelling requires moving beyond traditional ways of thinking applied in typical school problems to include contextual and critical analysis
Philosophical inquiry	Philosophical inquiry incorporates a range of thinking skills in identifying hidden assumptions, identifying alternative courses of action, and reflecting on conclusions drawn and claims made. Philosophical inquiry nurtures critical thinking dispositions
Systems thinking	Systems thinkers consider system boundaries, its components, interactions between components and different subsystems, and the emergent properties and behaviour of a system
Design-based thinking	Design-based thinking entails iterative processes usually involving problem scoping and idea generation, designing and creating, testing and reflecting, redesigning and recreating, and communicating
Adaptive and innovative thinking for learning innovation	Adaptive problem solvers have the flexibility, curiosity, and creativity to tackle novel problems, leading to learning innovation
	Learning innovation builds on core content to generate more powerful disciplinary knowledge and thinking processes that can, in turn, be adapted and applied to subsequent challenging problems

## Ways of thinking in STEM-based problem solving

### Critical thinking

Although long recognised as a significant process in a range of fields, research on critical thinking in education, especially in primary education, has been limited (Aktoprak & Hursen, 2022). Critical thinking has long been associated with mathematical reasoning and problem solving, but their association remains under-theorized (Jablonka, 2020). Likewise, connections between critical thinking and design thinking have had limited attention largely due to their shared conceptual structures not being articulated (Ericson, 2022). As a twenty-first century skill, critical thinking is increasingly recognised as essential in STEM and mathematics education (Kollosche & Meyerhöfer, 2021) but is sadly lacking in many school curricula (Braund, 2021). As applied to STEM-based problem solving, critical thinking builds on inquiry skills (Nichols et al., 2019) and entails evaluating and judging problem situations including statements, claims, and propositions made, analysing arguments, inferring, and reflecting on solution approaches and conclusions drawn. Although critical thinking can contribute significantly to each of the other ways of thinking, its application is often neglected. For example, critical thinking is increasingly needed in design and design thinking, which play a key role in product development, environmental projects, and even in forms of social interaction (as discussed in Sect. 3.3; Ericson, 2022).

#### Critical mathematical modelling

One rich source of problem experiences that foster critical thinking is that of modelling. The diverse field of mathematical modelling has long been prominent in the secondary years (e.g., Ärlebäck & Doerr, 2018) but remains under-researched in the primary years, especially in relation to its everyday applications. Effective engagement with social, political, and environmental issues through modelling and statistics demands critical thinking, yet such aspects are not often considered in school curricula (Jablonka, 2020). This is of particular concern, given the pressing need to tackle such issues in today’s world.

As noted in several publications, the interdisciplinary nature of mathematical modelling makes it ideal for STEM-based problem solving (English, 2016; Maass et al., 2019; Zawojewski et al., 2008). Numerous definitions of models and modelling exist in the literature (e.g., Blum & Leiss, 2007; Brady et al., 2015). For this article, modelling involves developing conceptual innovations in response to real-world needs; effective modelling requires moving beyond the conventional ways of thinking applied in typical school problems (Lesh et al., 2013) to include contextual and critical analysis.

Much has been written about the role of modelling during COVID-19. Mathematical models played a major role in grappling with COVID 19, but their projections were a source of controversy (Rhodes & Lancaster, 2020). There seems little appreciation of the critical nature of mathematical models in society (Barbosa, 2006) and how assumptions in the modelling process can sway decisions. STEM-based problem solving needs to incorporate not just modelling itself, but also critical mathematical modelling. Critiquing what a model yields, and what is learned, is of increasing social importance (Aguilar & Castaneda, 2021; Barbosa, 2006). Indeed, as Braund (2021) illustrated, the Covid-19 pandemic has revealed the urgent need for “critical STEM literacy” (p. 339)—an awareness of the complex and problematic interactions of STEM and politics, and a knowledge and understanding of the underlying STEM concepts and representations is essential:

There are two imperatives that emerge: first, that there is sufficient STEM literacy to negotiate the complex COVID-19 information landscape to enable personal decision taking and second, that this is accompanied by a degree of criticality so that politicians and experts are called to account (Braund, 2021, p. 339).

Kollosche (2021) shed further light on the lack of critical thinking in the media’s reporting on COVID, with his argument that “most newspaper reports were effective in creating the problems” because of their focus on ideal forms of mathematical concepts and modelling, without discussing the assumptions and methods behind the reported data. Of concern is that “mass media still fail to present scientific models and results in a way that allows for mathematical reflection and a critical evaluation of such information by citizens.”

Modelling experiences that draw on students’ cultural and community contexts (Anhalt et al., 2018; English, 2021a, 2021b; Turner et al., 2009) provide rich opportunities for critical thinking from a citizen’s perspective. Such opportunities can also assist students in appreciating that mathematics is not merely a means of calculating answers but is also a vehicle for social justice, where critical thinking plays a key role (Cirillo et al., 2016; Greer et al., 2007). In their studies of critical thinking in cultural and community contexts, Turner and her colleagues (Turner et al., 2009, 2021, 2022) explored culturally responsive, community-based approaches to mathematical modelling with elementary teachers and students. Using a range of authentic modelling contexts, Turner and her colleagues illustrated how students’ modelling processes generated a number of issues that required them to think critically about their lives and their lives within their community. In one activity, students were applying design thinking and processes as they redesigned their local park. They generated, for example, mathematical models to estimate how long children would have to wait to use the swings—this informed their decision that the park did not meet the needs of the community. This community-based modelling highlighted “ongoing negotiation between students’ experiences and intentions related to the community park, the constraints of the actual context, and the mathematical issues that arose” (Turner et al., 2009, p. 148).

Another example of modelling involving critical thinking in cultural and community contexts was implemented in a sixth-grade Cyprus classroom. Students were required to develop a model for their country to purchase water supplies from a choice of nearby nations (English & Mousoulides, 2011). Students were to consider travel distances, water price, available supply per week, oil tanker capacity, costs of water and oil, and quality of the port facilities in the neighbouring countries. The targeted model had to select the best option not only for the present but also for the future. Students’ models ranged from a basic form, where port facilities and water supply were ignored, to more sophisticated models, where all factors were integrated, with carbon emissions also considered. One of the student groups who took into account environmental factors commented, “It would be better for the country to spend a little more money and reduce oil consumption. And there are other environmental issues, like pollution of the Mediterranean Sea.” The more sophisticated models reflected systems thinking (Sect. 3.2), where the impact of partial factors such as oil consumption on the whole domain (ocean ecosystems and community) was also considered.

#### Philosophical Inquiry

One underrepresented means of fostering critical thinking in mathematical and STEM-based problem solving is through philosophical inquiry (Calvert et al., 2017; English, 2013, 2022; Kennedy, 2012; Mukhopadhyay & Greer, 2007). Such inquiry encourages a range of thinking skills in identifying hidden assumptions, determining alternative courses of action, and reflecting on conclusions drawn and claims made. Several studies have shown how engaging children in communities of philosophical inquiry nurtures critical thinking dispositions, which become both a goal and a method (Bezençon, 2020; Daniel et al., 2017; Lipman, 2003, 2008). At the same time, philosophical inquiry can lead to “conceptual deepening” (Bezençon, 2017), where analysis of mathematical and related STEM concepts as they apply beyond the classroom can be fostered. Given the increased societal awareness of mathematics and STEM in recent years, philosophical inquiry can be a powerful tool in enhancing students’ understanding and appreciation of how these disciplines shape societies. At the same time, philosophical inquiry can stimulate consideration of ethical issues in the applications of these disciplines (Bezençon, 2020). For example, Mukhopadhyay and Greer (2007) indicated how mathematics education should “convey the complexity of mathematical modeling social phenomena and a sense of what demarcates questions that can be answered by empirical evidence from those that depend on value systems and world-views” (p. 186).

A comprehensive review by O’Reilly et al. (2022) identified pedagogical approaches to scaffolding early critical thinking skills including inquiry-based teaching using classroom dialogue or questioning techniques. Such techniques include philosophical inquiry and encouraging children to construct, share, and justify their ideas regarding a task or investigation. Other opportunities for philosophical inquiry include group problem solving, peer sharing of created models, and facilitating critical and constructive peer feedback. For example, Gallagher and Jones (2021) reported on integrating mathematical modelling and economics, where beginning teachers were presented with a task involving a problematic community issue following a school shooting. In such cases, numerous courses of action are typically proposed for addressing the problem. Not surprisingly, various community opinions exist on such proposals, giving rise to valuable contexts for philosophical inquiry and critical modelling, where data and their sources are carefully analysed. With the escalation of statistical data from the mass media, it is imperative to commence the foundations of critical and philosophical thinking early. Students’ skills in asking critical questions as they work with data in constructing and improving a model, reflect on what their models convey, consider consequences of their models, and justify and communicate their conclusions require nurturing throughout school (Gibbs & Young Park, 2022).

### Systems thinking

Systems thinking cuts across the STEM disciplines as well as many other fields outside education. It is considered a key component of “critical thinking and problem solving” in *21st Century Learning* (P21, 2015) and is often cited as a “habit of mind” in engineering education (e.g., Lippard et al., 2018; Lucas et al., 2014). Numerous definitions exist for systems thinking (e.g., Bielik et al., 2022; Damelin et al., 2017; Jacobson & Wilenski, 2022), with Bielik et al. (2022) identifying such thinking as the ability to “consider the system boundaries, the components of the system, the interactions between system components and between different subsystems, and emergent properties and behaviour of the system” (p. 219). In more basic terms, Shin et al. (2022) refer to systems thinking as “the ability to understand a problem or phenomenon as a system of interacting elements that produces emergent behavior” (p. 936).

Systems thinking interacts with the other thinking forms including those displayed in Fig. 1, as well as computational thinking (Shin et al., 2022), critical thinking (Curwin et al., 2018) and mathematical thinking more broadly (Baioa & Carreira, 2022). Systems thinking is considered especially important in conceptualizing a problematic situation within a larger context and in perceiving problems in new and different ways (Stroh, 2018). Of special relevance to today’s world is the realisation that perfect solutions do not exist and the choices one makes in applying systems thinking will impact on other parts of the system (Meadows, 2008). What is often not considered in today’s complex societies—at least not to the extent required—is that we live in a world of intrinsically linked systems, where disruption in one part will reverberate in others. We see so many instances where particular courses of action are advocated or mandated in societal systems, while the impact on sub-components is perilously ignored. Examples are evident in many nations’ responses to COVID-19, where escalating lockdowns impacted economies and communities, whose demands had to be balanced against purely epidemiological factors. The reverberations of such actions stretch far and wide over long periods. Likewise, the various impacts of current climate actions are frequently ignored, such as how the construction of vast areas of renewable resources (e.g., wind turbines) can have deleterious effects on the surrounding environments including wildlife.

Despite its centrality across the STEM domains, systems thinking is almost absent from mathematics education (Curwin et al., 2018). This is despite claims by many researchers that modelling, systems thinking, and associated thinking processes should be significant components of students’ education (Bielik et al., 2022; Jacobson & Wilenski, 2022). Indeed, systems thinking is featured prominently in the US *A Framework for K-12 Science Education* (NRC, 2012) and the *Next Generation Science Standards* (NGSS Lead States, 2013), and has received considerable attention in science education (e.g., Borge, 2016; Hmelo-Silver et al., 2017; York et al., 2019) and engineering education (e.g., Lippard & Riley, 2018; Litzinger, 2016).

Given the complexity of systems thinking in today’s world, and the diverse ways in which it is applied, students require opportunities to experience how systems thinking can interact with other forms such as design thinking and critical thinking (Curwin et al., 2018; Shin et al., 2022). Such interactions occur in many popular STEM investigations including one in which fifth-grade students designed, constructed, and experimented with a loaded paper plane (paper clips added) in determining how load impacts on the distance travelled (English, 2021b). Students observed that changing one design feature (e.g., wingspan or load position) unsettles some other features (e.g., fuselage depth decreases wingspan). The investigation yielded an appreciation of the intricacy of interactions among a system’s components, and their scarcely predictable mutual effects.

Other studies have revealed how very young children can engage in basic systems thinking within STEM contexts. For example, Feriver et al. (2019) administered a story reading session to individual 4- to 6-year-old children in preschools in Turkey and Germany, and then followed this with individual semi-structured interviews about the story. The reading session was based on the story book, “The Water Hole” (Base, 2001), which draws on basic concepts of systems within an ecosystem context. Their study found the young children to have some complex understanding of systems thinking in terms of detecting obvious gradual changes and two-step domino and/or multiple one-way causalities, as well as describing the behaviour of a balancing loop (corrective actions that try to reduce the gap between a desired level or goal and the actual lever, such as temperature and plant growth). However, the children understandably experienced difficulties in several areas, in which even adults have problems. For example, detecting a reinforcing loop, identifying unintended consequences, detecting hidden components and processes, adopting a multi-dimensional perspective, and predicting how a system would behave in the future were problematic for them. In another study, Gillmeister (2017) showed how preschool children have a more complex understanding of systems thinking than previously claimed. Their ability to utilize simple systems thinking tools, such as stock-flow maps, feedback loops and behaviour over time graphs, was evident.

Although the research has not been extensive, current findings indicate how we might capitalise on the seeds of early systems thinking across the STEM fields. With the pervasive nature of systems thinking, it is argued that its connections to other forms of thinking across STEM should be nurtured (cf., Svensson, 2022). This includes links to design-based thinking where designed products, for example, operate “within broader systems and systems of systems” (Buchanan, 2019).

### Design-based thinking and STEM-based problem solving

Design-based thinking plays a major role in complex problem solving, yet its contribution to mathematics learning has been largely ignored, especially at the primary levels. Design is central in technology and engineering practice (English et al., 2020; Guzey et al., 2019), from bridge construction through to the development of medical tools. Design contributes to all phases of problem solving and drives students toward innovative rather than predetermined outcomes (Goldman & Zielezinski, 2016). As applied to STEM education, design thinking is generally viewed as a set of iterative processes of understanding a problem and developing an appropriate solution. It includes problem scoping, idea generation, systems thinking, designing and creating, testing and reflecting, redesigning and recreating, and communicating. Both learning *about* design and learning *through* design can help students develop more informed analytical approaches to mathematical and STEM-based problem solving (English et al., 2020; Strimel et al., 2018).

Learning *about* design in solving complex problems emphasises design thinking processes per se, that is, students learn about design itself. Design thinking as a means for learning about design is not as prevalent in integrated STEM education. Although learning through and about design should not be divorced during problem solving (van Breukelen et al., 2017), one learning goal can take precedence over the other. Both require greater attention, especially in today’s design-focused world (Tornroth & Wikberg Nilsson, 2022).

A plurality of solutions of varying levels of sophistication is possible in applying design thinking processes, so students from different achievement levels can devise solutions (Goldman & Kabayadondo, 2017)—and research suggests that lower-achieving students benefit most (Chin et al., 2019). The question of how best to develop design learning across grades K-12, however, has not received adequate attention. This is perhaps not surprising given that comparatively few studies have investigated the design thinking of grades 6–12 and even fewer across the elementary years (Kelley & Sung, 2017; Strimel et al., 2018).

Also overlooked is how design-based thinking can foster concept development, that is, how it can foster “learning while designing”, or generative learning (English et al., 2020). Design-based thinking provides natural opportunities to develop understanding of the required STEM concepts (Hjalmarson et al., 2020). As these authors pointed out, when students create designs, they represent models of their thinking. When students have to express their conceptual understanding in the form of design, such as an engineering designed product, their knowledge (e.g., of different material properties) is tested. Studies have revealed such learning occurs when elementary and middle school students design and construct various physical artifacts. For example, in a fifth-grade study involving designing and building an optical instrument that enabled one to see around corners, King and English (2017) observed how students applied and enriched their understanding of scientific concepts of light and how it travels through a system, together with their mathematical knowledge of geometric properties, angles and measurement.

In a study in the secondary grades (Langman et al. (2019), student groups completed modules that included a model-eliciting activity (Lesh & Zawojewski, 2007) involving iterative design thinking processes. Students were to interpret, assess, and compare images of blood vessel networks grown in scaffolds, develop a procedure or tool for measuring (or scoring) this vessel growth, and demonstrate how to apply their procedure to the images, as well as to any image of a blood vessel network. The results showed how students designed a range of mathematical models of varying levels of sophistication to evaluate the quality of blood vessel networks and developed a knowledge of angiogenesis in doing so.

In another study implemented in a 6th-grade classroom, students were involved in both learning about and learning through design in creating a new sustainable town with the goal of at least 50% renewable energy sources. The students were also engaged in systems thinking, as well as critical thinking, as they planned, developed, and critically analysed the layout and interactions of different components of their town system (e.g., where to place renewable energy sources to minimise impact on the residences and recreational areas; where to situate essential services to reach different town components). Students also had to consider the budgetary constraints in their town designs. Their learning about design was apparent in their iterative processes of problem scoping, idea generation and modification, balancing of benefits and trade-offs (dealing with system subcomponents), critical reflections on their designs, and improvements of the overall town design. Learning though design was evident as students displayed content knowledge pertaining to renewable energy and community needs, applied spatial reasoning in positioning town features, and considered budgetary constraints in reaching their “best” design.

Engineering design processes form a significant component of design thinking and learning, with foundational links across the STEM disciplines including mathematical modelling. Engineering design-based problems help students appreciate how they can apply different ideas and approaches from the STEM disciplines, with more than one outcome possible (Guzey et al., 2019). Such design thinking engages students in interpreting problems, developing initial design ideas, selecting and testing a promising design, analysing results from their prototype solution, and revising to improve outcomes.

It is of concern that limited attention has been devoted to engineering design-based thinking in mathematics and the other STEM disciplines in the primary grades. This is despite research indicating how very young children can engage in design-based thinking when provided with appropriate opportunities (e.g., Cunningham, 2018; Elkin et al., 2018). Elkin et al., for example, used robotics in early childhood classrooms to introduce foundational engineering design thinking processes. Their study illustrated how these young learners used engineering design journals and engaged in design thinking as they engineered creative solutions to challenging problems presented to them. It seems somewhat paradoxical that foundational engineering design thinking is a natural part of young children’s inquiry about their world, yet this important component has been largely ignored in these informative years.

In sum, design-based thinking is a powerful and integrative tool for mathematical and STEM-based problem solving and requires greater focus in school curricula. Both learning about and through design have the potential to improve learning and problem solving well beyond the school years (Chin et al., 2019). At the same time, design-based thinking, together with the other ways of thinking, can contribute significantly to learning innovation (English, 2018).

### Adaptive and innovative thinking for learning innovation

**Fig. 2:**
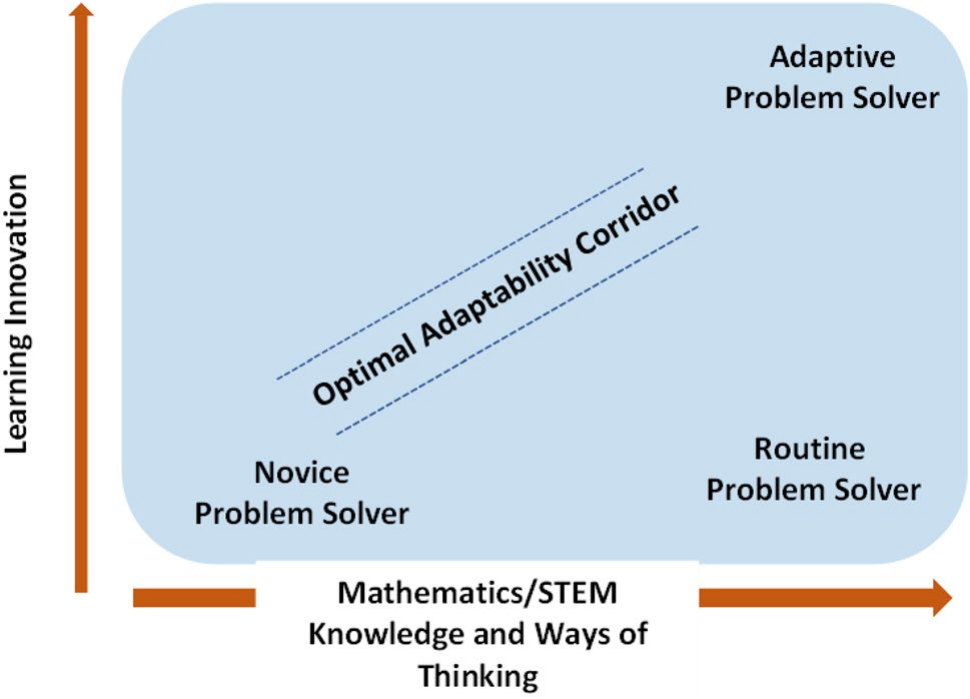
Learning innovation

Disruption is rapidly becoming the norm in almost all spheres of life. The recent national and international upheavals have further stimulated disruptive thinking (or disruptive innovation), where perspectives on commonly accepted (and often inefficient) solutions to problems are rejected for more innovative approaches and products. Given the pressing need for problem solvers capable of developing new and adaptable knowledge, rather than applying limited simplistic procedures or strategies, we need to foster what I term, *learning innovation* (English, 2018; Fig. 2). Such learning builds on core content to generate more powerful disciplinary knowledge and thinking processes that can, in turn, be adapted and applied to solving subsequent challenging problems.

Figure 2 (adapted from McKenna, 2014) illustrates how learning innovation can be fostered through the growth of mathematical and STEM knowledge, together with STEM ways of thinking. The *optimal adaptability corridor* represents the growth of mathematical and STEM-based problem solving from *novice* to *adaptable solver*. Adaptable problem solvers (Hatano & Oura, 2003) have developed the flexibility, curiosity, and creativity to tackle novel problems—skills that are needed in jobs of the future (Denning & Noray, 2020; OECD, 2019). Without the disciplinary knowledge, ways of thinking, and engagement in challenging but approachable problems, a student risks remaining merely a routine problem solver—one who is skilled solely in applying previously taught procedures to solve sets of familiar well-worn problems, as typically encountered at school. Learning innovation remains a challenging and unresolved issue across curriculum domains and is proposed as central to dealing effectively with disruption.

Particularly rich experiences for developing learning innovation involve interdisciplinary modelling, incorporating critical mathematical modelling (e.g., Hallström and Schönborn, 2019; Lesh et al., 2013). As noted, a key feature of model-eliciting experiences is the affordances they provide all students to exhibit “extraordinary abilities to remember and transfer their tools to new situations” (Lesh et al., 2013, p. 54). Modelling enables students to apply more sophisticated STEM concepts and generate solutions that extend beyond their usual classroom problems. Such experiences require different ways of thinking in problem solving as they deal with, for example, conflicting constraints and trade-offs, alternative paths to follow, and various tools and representations to utilise. In essence, interdisciplinary modelling may be regarded as a way of *creating* STEM concepts, with modeling and concept development being highly interdependent and mutually supportive (cf., Lesh & Caylor, 2007).

## Concluding points

The ill-defined problems of today, coupled with unexpected disruptions across all walks of life, demand advanced problem-solving by all citizens. The need to update outmoded forms of problem solving, which fail to take into account increasing global challenges, has never been greater (Cowin, 2021). The ways-of-thinking framework has been proposed as a powerful means of enhancing problem-solving skills for dealing with today’s unprecedented game-changers. Specifically, critical thinking (including critical mathematical modelling and philosophical inquiry), systems thinking, and design-based thinking are advanced as collectively contributing to the adaptive and innovative skills required for problem success. It is argued that the pinnacle of this framework is learning innovation, which can be within reach of all students. Fostering students’ agency for developing learning innovation is paramount if they are to take some control of their own problem solving and learning (English, 2018; Gadanidis et al., 2016; Roberts et al., 2022).

Establishing a culture of empowerment and equity, with an asset-based approach, where the strengths of all students are recognised, can empower students as learners and achievers in an increasingly uncertain world (Celedón-Pattichis et al., 2018). Teacher actions that encourage students to express their ideas, together with a program of future-oriented mathematical and STEM-based problems, can nurture students’ problem-solving confidence and dispositions (Goldman & Zielezinski, 2016; Roberts et al., 2022). In particular, mathematical and STEM-based modelling has been advocated as a rich means of developing multiple ways of thinking that foster adaptive and innovative learners—learners with a propensity for developing new knowledge and skills, together with a willingness to tackle ill-defined problems of today and the future. Such propensity for dealing effectively with STEM-based problem solving is imperative, beginning in the earliest grades. The skills gained can thus be readily transferred across disciplines, and subsequently across career opportunities.

## Data Availability

The data sets generated during and/or analysed during the current study are available from the corresponding author on reasonable request.
